# Leveraging dynamic serum uric acid trajectories for risk stratification in hospitalized HFpEF patients

**DOI:** 10.3389/fnut.2026.1802796

**Published:** 2026-06-08

**Authors:** Ziyang Bao, Xiaowei Liu, Xiaoqing Mao, Dongmin Zhang, Hongfeng Jin, Changqing Du, Yimin Tang, Xiaohong Xu

**Affiliations:** 1 Department of Nephrology, Hangzhou TCM Hospital Affiliated to Zhejiang Chinese Medical University, Hangzhou, Zhejiang, China; 2 Department of Cardiology, Zhejiang Hospital, Hangzhou, Zhejiang, China; 3 Department of Cardiology, QuZhou KeCheng People's Hospital, Quzhou, Zhejiang, China

**Keywords:** dynamic trajectories, heart failure with preserved ejection fraction, metabolic syndrome, risk stratification, serum uric acid

## Abstract

**Background:**

Single-timepoint SUA measurement provides limited prognostic insight in acute heart failure with preserved ejection fraction (HFpEF). This study assessed whether dynamic in-hospital SUA trajectories (using admission and pre-discharge measurements) are more strongly associated with long-term outcomes than static single-timepoint measures.

**Methods:**

The cohort consisted of 4,164 acute HFpEF patients, drawn from the China Heart Failure Center Registry. The primary outcome was a major adverse cardiovascular events (MACE). SUA at admission and pre-discharge was analyzed as continuous using Cox regression. Patients were categorized to four dynamic trajectory groups according to admission and pre-discharge SUA (cut-off 7 mg/dL): persistently normal (N-N), escalating (N-H), de-escalating (H-N), and persistently elevated (H-H). The optimal prognostic SUA cut-off was determined by ROC analysis.

**Results:**

Over a median of 25.2 months, 900 patients (21.6%) experienced the primary outcome. Multivariable Cox regression showed that pre-discharge SUA (HR 1.23, 95% CI 1.18–1.29, *p* < 0.001), but not admission SUA (HR 1.04, 95% CI 0.98–1.10, *p* = 0.17) was independently associated with MACE. Relative to the N-N group, dynamic trajectory analysis revealed significantly increased risk for the N-H group (adjusted HR 1.56, 95% CI 1.02–2.40, *p* = 0.041) and the H-H group (adjusted HR 1.61, 95% CI 1.10–2.34, *p* = 0.014), whereas the H-N group did not show a significant difference. The AUC of pre-discharge SUA for discriminating MACE risk was 0.70, with 84.3% sensitivity and 45.9% specificity at the 5.6 mg/dL threshold derived from ROC analysis. Subgroup and sensitivity analyses confirmed the robustness of these associations (all *p* for interaction >0.05 for most subgroups; results unchanged after excluding urate-lowering therapy or using multiple imputation).

**Conclusion:**

In hospitalized HFpEF patients, dynamic SUA trajectories are more strongly associated with MACE than single measurements. Identifying high-risk patterns prior to discharge may inform risk stratification and generate the hypothesis that nutritional or other modulatory strategies targeting SUA trajectories could be tested in future prospective studies to determine whether they improve outcomes.

## Introduction

Hospital readmissions and mortality following acute hospitalization remain a major challenge for healthcare systems globally, driving the search for modifiable risk factors and scalablemonitoring strategies (1). This is especially salient for heart failure with preserved ejection fraction (HFpEF), which representing close to half of total heart failure (HF) patients and imposes a substantial burden due to its poor prognosis and high recurrence rates (1, 2). Data from large-scale Chinese HF center registries indicate a 1-year HF readmission rate of 13.6% (3), 30-day, 1-year, and 3-year mortality rates of 2.0, 11.9 and 25.4%, respectively (4).

HFpEF pathophysiology is marked by multifaceted and heterogeneous mechanisms (5), involving systemic inflammation, neurohormonal activation, and metabolic dysregulation. The metabolic dimension, often termed the “cardiometabolic HFpEF” phenotype (6), is largely fueled by unhealthy lifestyles and the rising prevalence of metabolic comorbidities, such as insulin resistance. Despite the advent of therapies such as angiotensin receptor-neprilysin inhibitor (ARNI), finerenone, and sodium-glucose cotransporter-2 inhibitor (SGLT-2i), which have improved care (7), a significant unmet need persists for simple tools to stratify risk and guide management, especially after an acute hospitalization. The identification of robust biomarkers that are reliably associated with clinical outcomes is therefore important.

In this metabolic context, serum uric acid (SUA), a key component and a biomarker of this dysmetabolic state, represents a promising and readily accessible biomarker associated with prognosis (8–13). Beyond its association with gout, hyperuricemia is mechanistically linked to cardiovascular progression by the promotion of endothelial dysfunction, systemic inflammation and oxidative stress (8). This is particularly relevant in HFpEF, where a recent published meta-analysis confirmed that elevated SUA levels conferred a significantly elevated risk, raising all-cause death by 21%, HF hospitalization by 61%, and cardiovascular mortality by 71% (9). Further corroborating this, latest evidence indicates that in the HFpEF cohort, each 1 mg/dL increase in SUA corresponds to a 4% rise in major adverse cardiovascular events (MACE) risk (10). Of note, achieving a reduction in SUA of ≥1.0 mg/dL in this population has been linked to improved survival (11).

Despite this compelling association, critical translational gaps persist. First, the prognostic value of SUA has been defined almost exclusively by single-timepoint measurements, typically at hospital admission (14, 15). This static approach overlooks the clinically informative fluctuations that occur during and after acute hospitalization—driven by factors such as intravenous diuresis, hemodynamic changes, nutritional status, inflammation resolution, and early response to guideline-directed therapy (13). A single measurement cannot distinguish transient abnormalities from sustained elevations, nor capture the direction or magnitude of change. Tracking in-hospital SUA trajectories (reflecting real-time shifts in metabolic and nutritional homeostasis) could capture these changes and potentially reveal evolving risk earlier than any isolated measurement (16). However, although initial studies suggest this dynamic approach may be more valuable (16), it remains unexamined in large, dedicated HFpEF cohorts. Second, key questions regarding clinical application—including the optimal prognostic cut-off value and whether therapeutic SUA reduction improves hard endpoints—remain subjects of active debate (8, 11, 17).

In this study, we therefore evaluated both static and dynamic in-hospital SUA trajectories in a large Chinese HFpEF cohort to determine whether dynamic patterns provide superior risk stratification compared with single measurements, and to generate hypothesis-driven insights for future interventional studies.

## Materials and methods

### Study population

The CCA Database-HF serves as a Chinese national multicenter registry that incorporates data from hundreds of major medical institutions across China, focusing on HF patients (4, 5). For this retrospective analysis, we selected data from our institutional center within the CCA Database-HF. Patients hospitalized for an acute episode of HFpEF between January 2020 and December 2024 were enrolled in our cohort. This study sought to examine the role of both static SUA levels and their in-hospital fluctuation on post-discharge clinical adverse events.

Patients were enrolled only if they fulfilled the current guideline-based HFpEF diagnostic criteria (7), comprising: ① HF-related clinical features; ② a preserved ejection fraction (EF), defined as EF ≥ 50%; ③ elevated natriuretic peptides: defined as B-type natriuretic peptide (BNP) ≥ 35 pg./mL or N-terminal pro-BNP (NT-proBNP) ≥ 125 pg./mL (sinus rhythm) and BNP ≥ 105 pg./mL or NT-proBNP ≥365 pg./mL (atrial fibrillation); ④ two time points of complete SUA measurements were defined: at admission (within 24 h) and before discharge (within 48 h).

Key exclusion criteria encompassed: ① specific cardiac conditions that could solely account for HF (e.g., severe primary valvular disease, constrictive pericarditis, congenital heart disease); ② a recent history (within 30 days) of myocardial infarction, stroke or major cardiac surgery; ③ end-stage renal failure, including those with an estimated glomerular filtration rate (eGFR) < 15 mL/min/1.73 m^2^ or those receiving renal replacement therapy; ④ active malignancy or a limited life expectancy (below 1 year); and ⑤ critical missing data in essential baseline characteristics or follow-up information.

### Outcomes and follow-up

This study defined its primary outcome—termed MACE—as a composite of the first event of HF rehospitalization or all-cause death following discharge. As secondary endpoints, HF rehospitalization and all-cause death were also evaluated individually. HF rehospitalization was defined as a hospital readmission necessitated by the worsening of HF symptoms and signs (e.g., dyspnea, edema). All-cause death refers to mortality from any cause.

Follow-up was conducted until August 2025. According to the HF center study protocol (5), trained physicians and nurses from participating hospitals contacted patients via telephone or clinical visits at scheduled intervals after discharge: 1 week (±1 week), 1 month (±14 days), 3 months (±30 days), 1 year (±90 days), and annually (±90 days) thereafter, to collect clinical outcome data.

### Definition of SUA trajectory groups

This study focused on static SUA levels and their dynamic changes during hospitalization as the primary exposure variable. For static SUA analysis, levels measured at admission, pre-discharge and ΔSUA were included as continuous variables to examine their association with MACE. To capture dynamic changes, patients were classified to four distinct trajectories using SUA levels at admission and pre-discharge—a pragmatic simplification, as true trajectory analysis requires serial measurements—with clinical threshold of 7.0 mg/dL (18): the persistently normal group (normal-normal, N-N group), the escalation group (normal-high, N-H group), the de-escalation group (high-normal, H-N group), and the persistently elevated group (high-high, H-H group). The 7.0 mg/dL clinical threshold for hyperuricemia is widely accepted; in HFpEF, it affects 49–57% of patients and is independently associated with increased risks of all-cause death and HF readmission (19, 20).

### Data collection

From the database, comprehensive baseline information were obtained, including demographic information, coexisting conditions [e.g., hypertension, diabetes, chronic kidney disease (CKD), coronary artery disease (CAD)], prior HF hospitalization within 12 months, admission vital signs, laboratory results (e.g., serum creatinine, BNP, SUA, hemoglobin), echocardiographic parameters, and medication use (including SGLT-2i, beta-blockers, ARNI, mineralocorticoid receptor antagonists, diuretics and urate-lowering therapy).

### Statistical analysis

Data are summarized as mean ± SD or median (IQR) for continuous measures, and as *n* (%) for categorical measures. The Mann–Whitney *U* test, Chi-square test or Student’s *t*-test were used for comparisons between groups, as applicable.

To determine the relationship of static SUA with MACE, admission SUA, pre-discharge SUA and ΔSUA were analyzed as continuous variables in multivariable Cox regression models. A univariate *p*-value of less than 0.1 served as the criterion for a variable’s entry into the multivariable Cox regression. We constructed the final model by forcing in established prognostic factors (age, sex, eGFR, BNP, etc.) and the key exposure variable (admission SUA, pre-discharge SUA and ΔSUA). Due to collinearity among SUA-related predictors, three separate Cox models were built to evaluate their independent associations with MACE (A: admission SUA; B: pre-discharge SUA; C: ΔSUA). Among the remaining candidates, we selected variables by drawing upon their clinical relevance and further refined using a backward stepwise elimination procedure with a statistical cut-off of *p* < 0.05. Multicollinearity was checked by calculating the variance inflation factor (VIF), with a threshold of 5. Receiver operating characteristic (ROC) analysis was performed to evaluate the discriminatory ability of pre-discharge SUA for identifying MACE. Subsequently, Cox regression analysis was operated based on the derived optimal SUA threshold.

For the dynamic SUA trajectories, survival curves were constructed via the Kaplan–Meier method, with differences assessed by the log-rank test. We employed Cox regression (both univariable and multivariable) to examine the relationship of SUA trajectory groups with MACE risk, reporting results in the form of HRs and 95% CIs. The multivariable models incorporated baseline covariates that differed significantly among the trajectory groups, including demographics, medical history, key laboratory parameters, and baseline medications.

To assess the incremental association of pre-discharge SUA and SUA trajectory groups with MACE over conventional clinical variables, we constructed three nested Cox regression models. Baseline model included covariates as in the primary model. Clinical + pre-discharge SUA model added pre-discharge SUA (continuous) to the Baseline model. Clinical + trajectory model added the four-category SUA trajectory variable (N-N, N-H, H-N, H-H) to the Baseline model. Model fit was compared using the likelihood ratio test.

Subgroup analyses were performed to evaluate the consistency of the association between pre-discharge SUA (as a continuous variable) and MACE across key clinical subgroups. The following categorical subgroup variables were predefined based on clinical relevance or established cut-offs: sex (male vs. female), age (<65 vs. ≥65 years), body mass index (BMI < 24 vs. ≥24 kg/m^2^); eGFR (< 60 vs. ≥60 mL/min/1.73 m^2^); AF (yes vs. no); diabetes (yes vs. no); HF hospitalization within the previous 12 months (yes vs. no); and loop diuretic use at discharge (yes vs. no). For each subgroup, a separate multivariable Cox regression model was fitted with pre-discharge SUA as the exposure, adjusting for the same set of covariates as in the primary model. To formally test for interaction, we included a product term between pre-discharge SUA (continuous) and each subgroup variable (categorical) in the fully adjusted Cox model. The *p*-value for interaction was derived from the Wald test of the product term. All interaction tests were performed without adjusting for multiple comparisons, given their exploratory nature.

To verify the key associations, sensitivity analyses included (1) repeating the Cox regressions after excluding patients on urate-lowering therapy at admission or discharge, and (2) addressing missing baseline covariates (5.1–18.4%) using complete case analysis as the primary approach and multiple imputation (5 imputed datasets, including all analytical variables) as a sensitivity analysis.

We performed the analyses using IBM SPSS Statistics 26.0 and R 4.3.1, defining statistical significance as a two-sided *p*-value < 0.05.

## Results

### Baseline characteristics of the cohort

**Figure 1:**
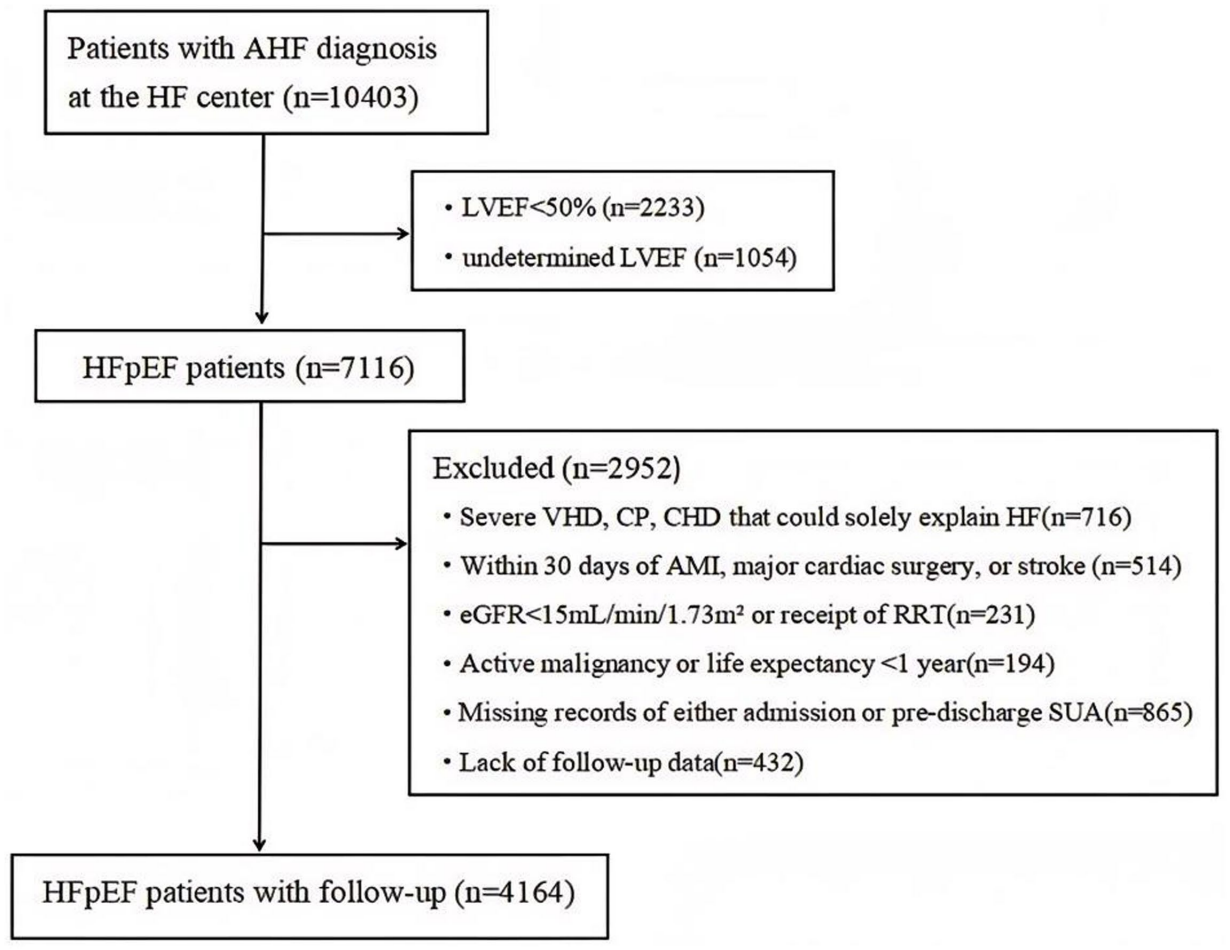
Flowchart depicting the participant selection process for the study. AHF, acute heart failure; HF, heart failure; LVEF, left ventricular ejection fraction; HFpEF, heart failure with preserved ejection fraction; VHD, valvular heart disease; CP, constrictive pericarditis; CHD, congenital heart disease; AMI, acute myocardial infarction; eGFR, estimated glomerular filtration rate; RRT, renal replacement therapy; SUA, serum uric acid.

Totally, 10,403 patients hospitalized for acute HF were initially recognized from our center’s HF database during the study period (January 2020–December 2024). Of these, 7,116 patients (68.4%) had a LVEF ≥ 50%. The final study cohort consisted of 4,164 HFpEF patients, following the preset eligibility criteria. The screening flowchart with specific exclusion reasons is presented in Figure 1.

Baseline characteristics for all included patients are detailed in Table 1. Briefly, the study population presented with a mean age of 71.0 ± 15.1 years, a mean BMI of 24.1 ± 3.3 kg/m^2^, and 56% were male. Comorbidities were common, with a mean Charlson Comorbidity Index of 3.0 ± 1.5. Prevalent conditions included hypertension (72.8%), CAD (62.1%), diabetes (31.6%), and CKD (23.2%). The mean SUA level at admission and pre-discharge was 6.2 ± 1.8 and 6.2 ± 1.7 mg/dL, respectively. At discharge, 1,264 patients (30.4%) met the criteria for hyperuricemia (SUA ≥ 7.0 mg/dL or ongoing urate-lowering therapy), among whom 421 patients (approximately one-third) were receiving urate-lowering therapy.

**Table 1:** Baseline characteristics for all cohort and SUA trajectory groups.

	Total population (*n* = 4,164)	N-N group (*n* = 2,806)	N-H group (*n* = 175)	H-N group (*n* = 628)	H-H group (*n* = 555)	Inter-group *p-*value
Age, years	71.0 ± 15.1	70.3 ± 14.4	74.4 ± 14.9	71.3 ± 16.4	73.5 ± 15.1	<0.001
Male sex, *n* (%)	2,327 (55.9%)	1,399 (49.9%)	99 (56.6%)	445 (70.9%)	384 (69.2%)	<0.001
Smoking	1,038 (24.9%)	702 (25.0%)	43 (24.6%)	166 (26.4%)	127 (22.9%)	0.57
BMI, kg/m^2^	24.1 ± 3.3	23.9 ± 3.2	23.9 ± 3.4	24.7 ± 3.3	24.8 ± 3.5	<0.001
Blood pressure, mmHg						
Systolic	136.3 ± 20.6	136.1 ± 20.3	135.7 ± 20.6	137.7 ± 21.3	135.9 ± 21.4	0.45
Diastolic	76.1 ± 13.9	75.9 ± 13.4	74.3 ± 15.8	77.5 ± 14.8	76.3 ± 14.8	0.057
HR, bpm	75.6 ± 15.7	75.3 ± 15.3	76.1 ± 18.5	77.3 ± 17.0	75.4 ± 15.4	0.071
Length of hospital stay, days, median (IQR)	5.6 (3.7, 8.8)	5.0 (3.2, 8.0)	7.0 (4.9, 11.0)	6.1 (3.9, 10.0)	5.8 (3.9, 9.1)	0.28
Comorbidities						
Charlson comorbidity index	3.0 ± 1.5	2.8 ± 1.4	3.5 ± 1.7	3.3 ± 1.6	3.5 ± 1.7	<0.001
Hypertension, *n* (%)	3,033 (72.8%)	1981 (70.6%)	130 (74.3%)	495 (78.8%)	427 (76.9%)	<0.001
CAD, *n* (%)	2,586 (62.1%)	1729 (61.6%)	109 (62.3%)	379 (60.4%)	369 (66.5%)	0.13
OMI, *n* (%)	474 (11.4%)	293 (10.4%)	27 (15.4%)	74 (11.8%)	80 (14.4%)	0.015
Coronary revascularization, *n* (%)	547 (13.1%)	345 (12.3%)	25 (14.3%)	68 (10.8%)	109 (19.6%)	<0.001
Diabetes, *n* (%)	1,314 (31.6%)	841 (30.0%)	63 (36.0%)	197 (31.4%)	213 (38.4%)	0.001
Gout, *n* (%)	170 (4.1%)	69 (2.5%)	9 (5.1%)	40 (6.4%)	52 (9.4%)	<0.001
AF, *n* (%)	1,463 (35.1%)	895 (31.9%)	87 (49.7%)	255 (40.6%)	226 (40.7%)	<0.001
Stroke, *n* (%)	593 (14.2%)	408 (14.5%)	26 (14.9%)	89 (14.2%)	70 (12.6%)	0.69
CKD, *n* (%)	967 (23.2%)	463 (16.5%)	70 (40.0%)	210 (33.4%)	224 (40.4%)	<0.001
Previous HF hospitalization within 12 months, *n* (%)	474 (11.4%)	252 (9.0%)	31(17.8%)	76 (12.1%)	115 (20.7%)	<0.001
Laboratory data						
Hemoglobin, mg/dL	127.0 ± 20.1	127.6 ± 18.6	119.2 ± 20.4	127.4 ± 23.1	126.0 ± 22.9	<0.001
eGFR, mL/min/1.73m^2^	60.5 ± 20.6	65.2 ± 18.2	51.1 ± 20.9	52.7 ± 21.3	48.3 ± 22.0	<0.001
BNP before discharge, pg./mL, median (IQR)	168.5 (76.0, 337.0)	138.0 (65.0, 278.0)	297.9 (126.3, 474.5)	222.0 (106.9, 401.0)	286.7 (125.7, 455.0)	<0.001
HbA1c, mmol/L	6.4 ± 1.2	6.4 ± 1.2	6.4 ± 1.3	6.3 ± 1.1	6.5 ± 1.1	0.78
SUA at admission, mg/dL	6.2 ± 1.8	5.3 ± 1.1	5.8 ± 1.1	8.3 ± 1.2	9.6 ± 1.3	<0.001
SUA before discharge, mg/dL	6.2 ± 1.7	5.5 ± 1.1	8.7 ± 1.3	5.9 ± 1.0	9.0 ± 1.6	<0.001
LDL-C, mmol/L	2.1 ± 0.9	2.2 ± 0.9	2.2 ± 1.0	2.2 ± 1.0	2.1 ± 0.9	0.22
Echocardiographic parameters						
LVEF (%)	62.9 ± 7.3	63.1 ± 7.3	61.6 ± 7.6	62.7 ± 7.4	62.2 ± 7.0	0.019
LVEDD, mm	47.4 ± 6.4	47.1 ± 6.3	47.8 ± 7.6	47.9 ± 6.3	48.4 ± 6.7	0.002
Therapy at discharge						
*β*-blocker, *n* (%)	2,420 (58.1%)	1,551 (55.3%)	125 (71.4%)	409 (65.1%)	335 (60.4%)	<0.001
ACEI/ARB/ARNI, *n* (%)	2,327 (55.9%)	1,532 (54.6%)	105 (60.0%)	391 (62.3%)	299 (53.9%)	0.002
ARNI, *n* (%)	1,324 (31.8%)	857 (30.5%)	58 (33.1%)	223 (35.5%)	186 (33.5%)	0.074
SGLT-2i, *n* (%)	962 (23.1%)	615 (21.9%)	47 (26.9%)	169 (26.9%)	131 (23.6%)	0.032
MRA, *n* (%)	1,386 (33.3%)	815 (29.0%)	97 (55.4%)	271 (43.2%)	203 (36.6%)	<0.001
Loop Diuretic, *n* (%)	1889(45.4%)	1,079 (38.5%)	127 (72.6%)	368 (58.6%)	315 (56.8%)	<0.001
Antiplatelet drugs, *n* (%)	2,250 (54.0%)	1,537 (54.8%)	100 (57.1%)	333 (53.0%)	280 (50.5%)	0.22
OACs, *n* (%)	1,448 (34.8%)	896 (31.9%)	85 (48.6%)	256 (40.8%)	211 (38.0%)	<0.001
Statin, *n* (%)	3,132 (75.2%)	2,128 (75.8%)	134 (76.6%)	463 (73.7%)	407 (73.3%)	0.47
Urate-lowering therapy, *n* (%)	421 (10.1%)	296 (10.5%)	18 (10.3%)	29 (4.6%)	78 (14.1%)	<0.001

SUA, serum uric acid; BMI, body mass index; HR, heart rate; CAD, coronary artery disease; OMI, old myocardial infarction; AF, atrial fibrillation; CKD, chronic kidney disease; COPD, chronic obstructive pulmonary disease; HF, heart failure; eGFR, estimated glomerular filtration rate; BNP, B-type natriuretic peptide; LDL-C, low density lipoprotein cholesterol; LVEF, left ventricular ejection fraction; LVEDD, left ventricular end-diastolic dimension; ACEI/ARB/ARNI, angiotensin converting enzyme inhibitor/angiotensin receptor blocker/angiotensin receptor neprilysin inhibitor; SGLT-2i, sodium glucose cotransporter-2 inhibition; MRA, mineralocorticoid recept antagonist; OACs, oral anticoagulants.

### Outcomes

#### Factors associated with MACE

Over a median of 25.2 months, the occurrence of endpoint events is summarized in Table 2. Among the 4,164 included patients, 900 (21.6%) experienced a MACE event, with an incidence of 13.2% (548 events) within the first year. HF rehospitalization occurred in 704 patients (16.9%) and all-cause death occurred in 280 patients (6.7%).

**Table 2:** Primary endpoint events and secondary endpoint events.

	Total population (*n* = 4,164)	N-N group (*n* = 2,806)	N-H group (*n* = 175)	H-N group (*n* = 628)	H-H group (*n* = 555)	Inter-group *p-*value
MACE	900 (21.6%)	458 (16.3%)	70 (40.0%)	136 (21.7%)	236 (42.5%)	<0.001
1-year MACE	548 (13.2%)	256 (9.1%)	47 (26.9%)	84 (13.4%)	161 (29.0%)	<0.001
HF rehospitalization	704 (16.9%)	412 (14.7%)	49 (28.0%)	105 (16.7%)	138 (24.9%)	<0.001
1-year HF rehospitalization	486 (11.7%)	253 (9.0%)	38 (21.7%)	80 (12.7%)	115 (20.7%)	<0.001
All-cause mortality	280 (6.7%)	75 (2.7%)	28 (16.0%)	39 (6.2%)	138 (24.9%)	<0.001
1-year all-cause mortality	113 (2.7%)	17 (0.6%)	15 (8.6%)	8 (1.3%)	73 (13.2%)	<0.001

MACE, major adverse cardiovascular events; HF, heart failure.

Baseline characteristics according to MACE status are presented in Table 3. Multivariable Cox regression analyses were performed with SUA modeled as continuous predictors in three separate models: admission SUA (Model A), pre-discharge SUA (Model B), and ΔSUA (Model C, pre-discharge minus admission). All models were adjusted for gender, age, AF, prior HF hospitalization, pre-discharge BNP, and eGFR, as well as other conventional risk factors (detailed in Table 3).

**Table 3:** Multivariate Cox regression analysis for MACE: comparison of admission SUA (Model A), pre-discharge SUA (Model B), and ΔSUA (Model C) as continuous predictors.

Variable	Model A (admission SUA)	Model B (pre-discharge SUA)	Model C (ΔSUA)
SUA-related predictor			
Admission SUA (per 1 mg/dL)	1.04 (0.98–1.10)	—	—
Pre-discharge SUA (per 1 mg/dL)	—	1.23 (1.18–1.29)**	—
ΔSUA (per 1 mg/dL increase)^†^	—	—	1.18 (1.14–1.22)**
Other covariates			
Gender (compare to women)	1.06 (0.87–1.30)	1.02 (0.84–1.24)	1.25 (1.01–1.55)*
Age	1.021 (1.002–1.040)*	1.02 (1.01–1.03)**	1.03 (1.02–1.04)**
Hypertension	0.98 (0.77–1.23)	0.99 (0.79–1.25)	0.90 (0.70–1.17)
CAD	1.10 (0.90–1.36)	1.09 (0.89–1.34)	1.03 (0.88–1.21)
Diabetes	1.13 (0.88–1.38)	1.18 (0.97–1.44)	1.27 (1.04–1.56)*
AF	1.30 (1.061–1.59)*	1.24 (1.02–1.52)*	1.43 (1.15–1.78)**
Previous HF hospitalization within 12 months	3.81 (2.99–4.43)**	3.45 (2.76–4.13)**	4.39 (2.31–6.81)**
Hemoglobin (per 1 g/dL)	1.00 (0.99–1.01)	1.001 (0.996–1.006)	1.001 (0.995–1.006)
eGFR (per 1 mL/min/1.73m^2^)	0.989 (0.983–0.995)**	0.991 (0.985–0.997)*	1.003 (1.003–1.004)**
Pre-discharge BNP (per 1 pg./mL)	1.002 (1.001–1.002)**	1.002 (1.001–1.002)**	1.001 (1.001–1.002)**
LDL-C (per 1 mol/L)	1.03 (0.85–1.20)	1.03 (0.93–1.15)	1.04 (0.93–1.17)
LVEF (per 1%)	1.00 (0.98–1.03)	1.01 (1.00–1.03)	1.00 (0.99–1.01)

^†^ΔSUA = pre-discharge SUA minus admission SUA; positive values indicate an increase during hospitalization. *p < 0.05, **p < 0.001.

In univariate analysis, all variables except gender were significantly associated with MACE (*p* < 0.001 for each; data not shown).

MACE, major adverse cardiovascular events; SUA, serum uric acid; CAD, coronary artery disease; AF, atrial fibrillation; eGFR, estimated glomerular filtration rate; BNP, B-type natriuretic peptide; LDL-C, low density lipoprotein cholesterol; LVEF, left ventricular ejection fraction.

Model B (pre-discharge SUA) demonstrated a significant and independent association with MACE (adjusted HR 1.23 per 1 mg/dL, 95% CI 1.18–1.29, *p* < 0.001). Model C (ΔSUA) also revealed a strong positive association (adjusted HR 1.18 per 1 mg/dL increase, 95% CI 1.14–1.22, *p* < 0.001), indicating that an increase in SUA during hospitalization is independently linked to higher MACE risk. In contrast, Model A (admission SUA) showed no significant association with MACE (HR 1.04, 95% CI 0.98–1.10, *p* = 0.17). The associations of the other covariates remained consistent across all models (Table 3).

#### Association of static SUA levels with MACE

**Figure 2:**
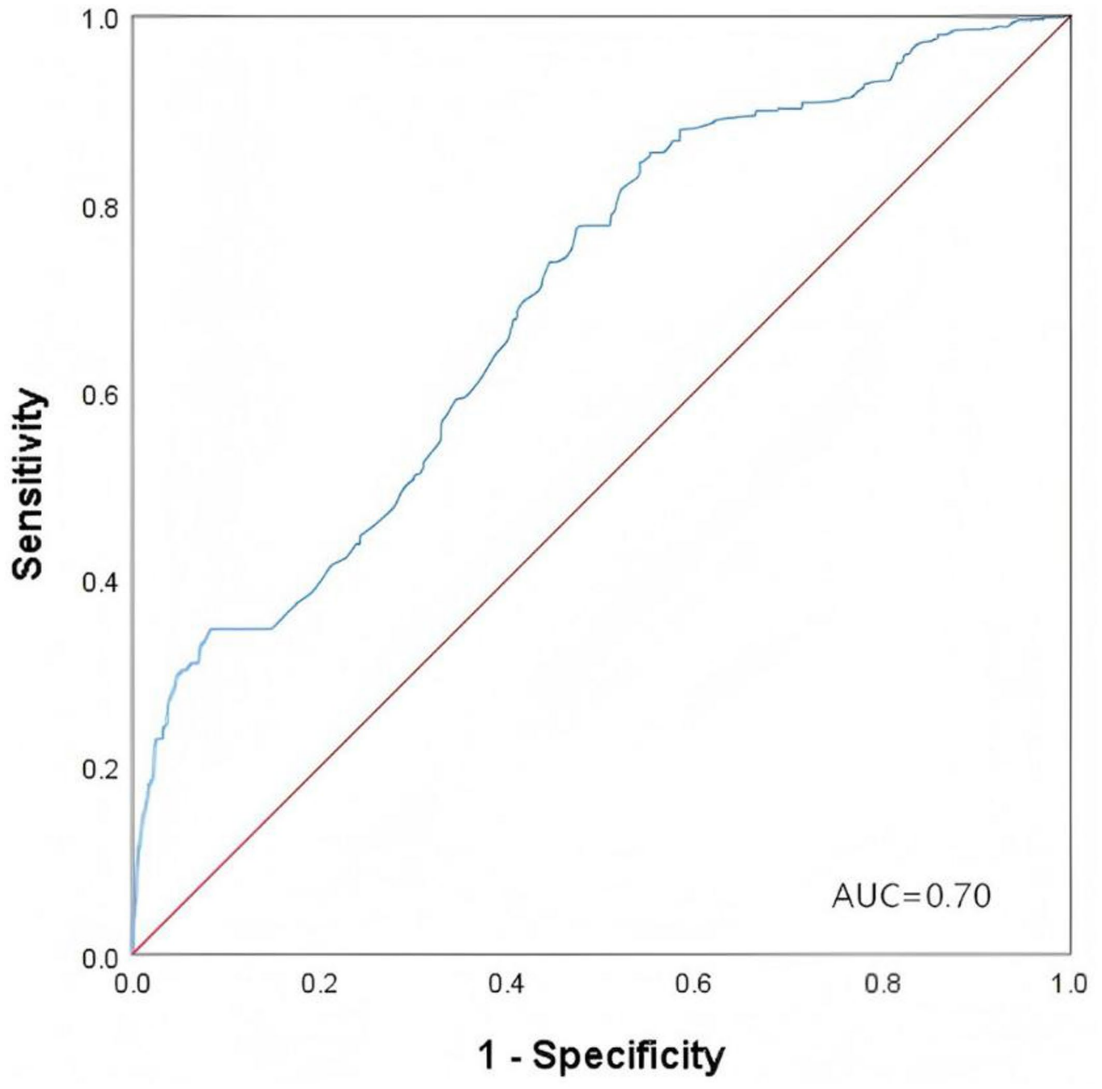
ROC curve of pre-discharge SUA for MACE in HFpEF. SUA, serum uric acid; MACE, major adverse cardiovascular events.

ROC curve analysis (Figure 2) revealed that pre-discharge SUA had modest discriminative ability for MACE (AUC = 0.70). At the optimal cut-off of 5.6 mg/dL, sensitivity was 84.3% and specificity 45.9%—adequate for screening but insufficient for standalone risk prediction.

Subsequently, multivariable Cox regression analyses were performed with pre-discharge SUA modeled both in continuous and binary form (using the 5.6 mg/dL cut-off). (The 7.0 mg/dL threshold for trajectory grouping follows the clinical definition of hyperuricemia, while this ROC-derived 5.6 mg/dL cut-off optimizes the discrimination of pre-discharge SUA for MACE; the two thresholds address different questions and are not comparable.) As presented in Table 4, after full adjustment for covariates, a significant and independent association persisted between elevated pre-discharge SUA levels and MACE risk, whether analyzed per unit increase as a continuous variable (adjusted HR 1.22, 95% CI 1.15–1.29, *p* < 0.001) or when compared as high versus low level using the cut-off (adjusted HR 2.37, 95% CI 1.78–3.16, *p* < 0.001).

**Table 4:** Multivariate Cox regression analysis for MACE according to pre-discharge SUA level.

	SUA as continuous variable		SUA as binary variable*	
	HR (95%CI)	*p*-value	HR (95%CI)	*p-*value
Model 1	1.47 (1.42–1.51)	<0.001	3.98 (3.32–4.78)	<0.001
Model 2	1.54 (1.48–1.60)	<0.001	3.72 (3.05–4.54)	<0.001
Model 3	1.41 (1.36–1.46)	<0.001	3.67 (3.00–4.78)	<0.001
Model 4	1.29 (1.22–1.37)	<0.001	2.51 (1.89–3.33)	<0.001
Model 5	1.22 (1.15–1.29)	<0.001	2.37 (1.78–3.16)	<0.001

^*^Compared to SUA < 5.6 mg/dL before discharge. Model 1: Unadjusted; Model 2: adjusted for demographic factors (age, gender, BMI); Model 3: Model 2 + adjusted for comorbidities (Hypertension, coronary revascularization, diabetes, AF, previous HF hospitalization within 12 months); Model 4: Model 3 + adjusted for laboratory parameters (Hemoglobin, eGFR, BNP before discharge, LVEF, LVEDD); Model 5: Model 4 + adjusted for medication history (β-blocker, ACEI/ARB/ARNI, SGLT-2i, MRA, loop diuretic use, urate-lowering therapy).

MACE, major adverse cardiovascular events; SUA, serum uric acid; BMI, body mass index; CAD, coronary artery disease; AF, atrial fibrillation; HF, heart failure; eGFR, estimated glomerular filtration rate, BNP, B-type natriuretic peptide; LVEF, left ventricular ejection fraction; LVEDD, left ventricular end-diastolic dimension; ACEI/ARB/ARNI, angiotensin converting enzyme inhibitor/angiotensin receptor blocker/angiotensin receptor neprilysin inhibitor; SGLT-2i, Sodium glucose cotransporter-2 inhibition; MRA, mineralocorticoid recept antagonist.

#### Association of dynamic SUA trajectories with MACE

To examine the effect of in-hospital SUA fluctuation on outcomes, patients were classified to four distinct trajectories on the basis of their admission and pre-discharge levels (see Methods). Table 1 summarizes the baseline characteristics and Table 2 presents the incidence of endpoint events across the four trajectory groups. In the N-N group, 458 of 2,806 patients (16.3%) experienced a MACE, with 412 (14.7%) hospitalized for HF and 75 (2.7%) dying. In contrast, the H-H group had substantially higher event rates: 236 of 555 patients (42.5%) had a MACE, 161 (29.0%) were rehospitalized for HF, and 138 (24.9%) died.

**Figure 3:**
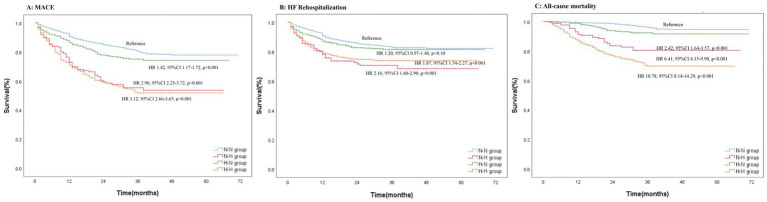
Kaplan–Meier curves in HFpEF among the four SUA trajectory groups. **(A)** Kaplan–Meier curves in HFpEF for MACE; **(B)** Kaplan–Meier curves in HFpEF for HF rehospitalization; **(C)** Kaplan–Meier curves in HFpEF for all-cause mortality. HFpEF, heart failure with preserved ejection fraction; SUA, serum uric acid; MACE, major adverse cardiovascular events; HF, heart failure.

Consistent risk stratification was observed across all endpoints. Kaplan–Meier analyses for MACE, HF rehospitalization, and all-cause mortality all demonstrated significant separation among the four SUA trajectory groups, with all log-rank *p-*values <0.001, among which the N-H and H-H groups consistently exhibited the worst event-free survival. The corresponding survival curves are presented in Figure 3.

Cox regression analysis showed significant associations between SUA trajectory groups and MACE (Table 5). In the unadjusted model, compared with the N-N group, a higher hazard of MACE was observed in the N-H (unadjusted HR 2.90, 95% CI 2.25–3.72, *p* < 0.001), H-N (unadjusted HR 1.42, 95% CI 1.17–1.72, *p* < 0.001), and H-H groups (unadjusted HR 3.12, 95% CI 2.66–3.65, *p* < 0.001). After full adjustment, the associations remained elevated in the N-H (adjusted HR 1.56, 95% CI 1.02–2.40, *p* = 0.041) and H-H groups (adjusted HR 1.61, 95% CI 1.10–2.34, *p* = 0.014), but were substantially attenuated and non-significant in the H-N group (adjusted HR 0.80, 95% CI 0.53–1.22, *p* = 0.30).

**Table 5:** Multivariate Cox regression analysis for MACE according to SUA trajectory groups.

	N-N group		N-H group		H-N group		H-H group	
	HR (95%CI)	*p-*value	HR (95% CI)*	*p-*value	HR (95% CI)*	*p-*value	HR (95% CI)*	*p-*value
Model 1	—	—	2.90 (2.25–3.72)	<0.001	1.42 (1.17–1.72)	<0.001	3.12 (2.66–3.65)	<0.001
Model 2	—	–	2.58 (2.00–3.32)	<0.001	1.32 (1.09–1.60)	<0.001	2.74 (2.34–3.22)	<0.001
Model 3	—	—	1.81 (1.35–2.42)	<0.001	1.22 (0.99–1.51)	0.062	2.01 (1.67–2.42)	<0.001
Model 4	—	—	1.45 (1.06–1.98)	0.021	0.91 (0.73–1.14)	0.43	1.74 (1.43–2.12)	<0.001
Model 5	—	—	1.56 (1.02–2.40)	0.041	0.80 (0.53–1.22)	0.30	1.61 (1.10–2.34)	0.014

*Compared to N-N group. Model 1: Unadjusted; Model 2: adjusted for demographic factors (age, gender, BMI); Model 3: Model 2 + adjusted for comorbidities (Hypertension, coronary revascularization, diabetes, AF, previous HF hospitalization within 12 months); Model 4: Model 3 + adjusted for laboratory parameters (Hemoglobin, eGFR, BNP before discharge, LVEF, LVEDD); Model 5: Model 4 + adjusted for medication history (*β*-blocker, ACEI/ARB/ARNI, SGLT-2i, MRA, loop diuretic use, urate-lowering therapy).

MACE, major adverse cardiovascular events; SUA, serum uric acid; BMI, body mass index; CAD, coronary artery disease; AF, Atrial Fibrillation; HF, heart failure; eGFR, estimated glomerular filtration rate, BNP, B-type natriuretic peptide; LVEF, left ventricular ejection fraction; LVEDD, left ventricular end-diastolic dimension; ACEI/ARB/ARNI, angiotensin converting enzyme inhibitor/angiotensin receptor blocker/angiotensin receptor neprilysin inhibitor; SGLT-2i, sodium glucose cotransporter-2 inhibition; MRA, mineralocorticoid recept antagonist.

#### Incremental association of pre-discharge SUA and trajectory groups with MACE

The Clinical + pre-discharge SUA model (−2LL = 4,172) showed a significant improvement in model fit compared with the Baseline model (−2LL = 4,219; likelihood ratio *χ*^2^ = 47, df = 1, *p* < 0.001). Similarly, the Clinical + trajectory model (−2LL = 4,170) also improved fit significantly (*χ*^2^ = 49, df = 1, *p* < 0.001). These results indicate that both single pre-discharge SUA and dynamic SUA trajectories provide incremental prognostic information beyond conventional clinical variables, as showed in Table 6.

**Table 6:** Likelihood ratio tests comparing three nested Cox models.

Model	−2LL	Likelihood ratio *χ*^2^	df	*p*-value
Baseline clinical model	4,219	—	—	—
+ Pre-discharge SUA	4,172	47	1	<0.001*
+ SUA trajectory groups	4,170	49	1	<0.001*

^*^The likelihood ratio tests compare Model B and Model C with the Baseline clinical model (adjusted for age, sex, BMI, hypertension, CAD, diabetes, AF, prior HF hospitalization, hemoglobin, eGFR, pre-discharge BNP, LVEF, LVEDD, β-blocker, ACEI/ARB/ARNI, SGLT-2i, MRA, loop diuretic, and ULT).

CAD, coronary artery disease; AF, atrial fibrillation; eGFR, estimated glomerular filtration rate; BNP, B-type natriuretic peptide; LVEF, left ventricular ejection fraction; LVEDD, left ventricular end diastolic dimension; ACEI/ARB/ARNI, angiotensin-converting enzyme inhibitor/angiotensin receptor blocker/angiotensin receptor neprilysin inhibitor; SGLT-2i, sodium-glucose cotransporter-2 inhibitor; MRA, mineralocorticoid receptor antagonist; ULT, urate-lowering therapy.

#### Subgroup analysis

**Figure 4:**
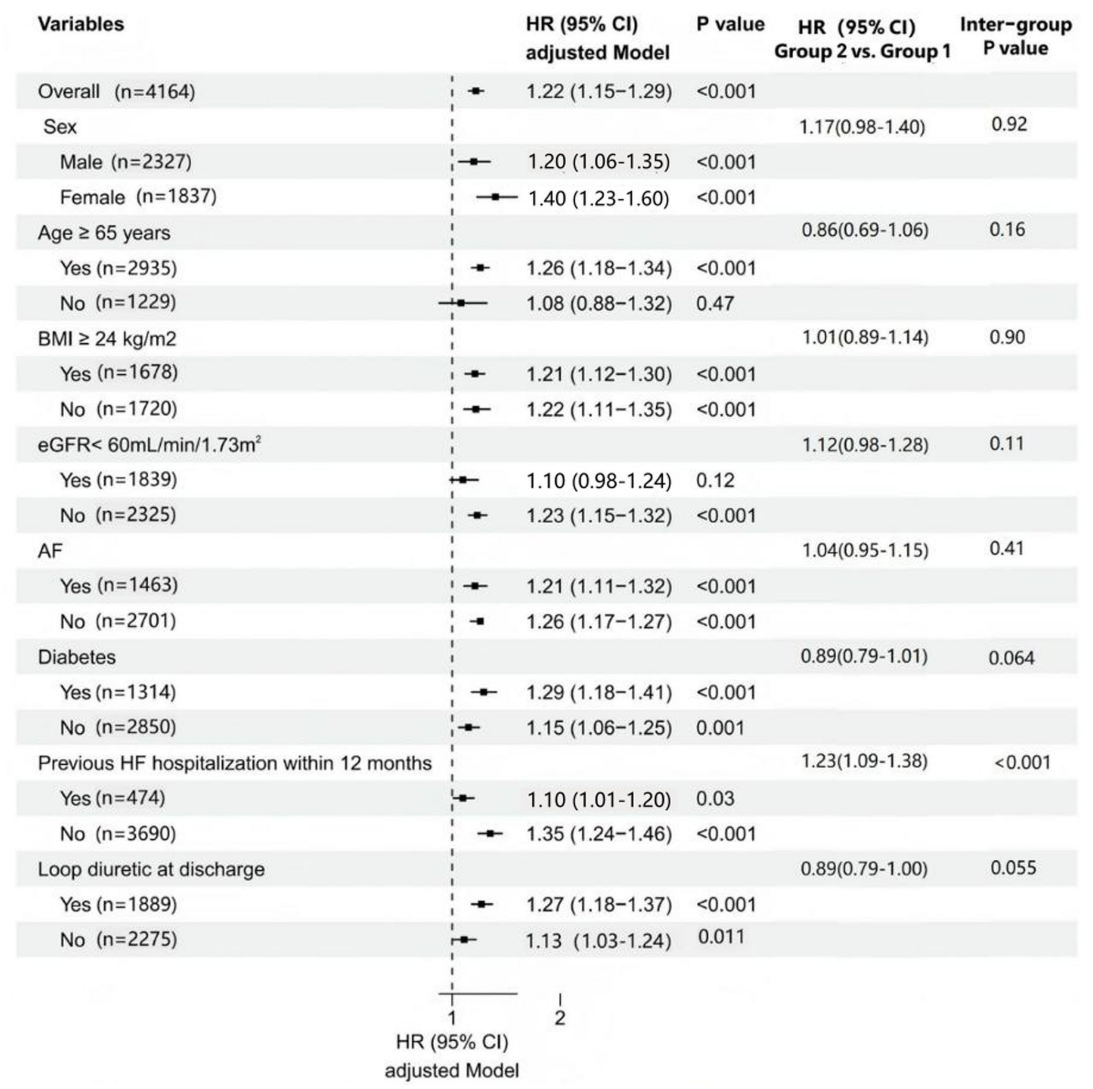
Subgroup analysis of pre-discharge SUA on MACE events. SUA, serum uric acid; MACE, major adverse cardiovascular events; B5/31/2026MI, body mass index; eGFR, estimated glomerular filtration rate; AF, atrial fibrillation; HF, heart failure.

As detailed in Figure 4, Subgroup analyses showed that the association between pre-discharge SUA and MACE was generally consistent across most subgroups, with no significant interactions observed for sex, BMI, diabetes, AF or loop diuretic use at discharge (all *p* for interaction >0.05). However, the association was more pronounced in patients without a history of HF hospitalization within the previous 12 months (HR 1.23, *p* for interaction <0.001) compared to their counterparts. In contrast, no significant associations were found in patients aged <65 years (adjusted HR 1.08, *p* = 0.47) or in those with CKD (adjusted HR 1.10, *p* = 0.12), whereas significant associations were observed in patients aged ≥65 years (HR 1.26, *p* < 0.001) and in those without CKD (HR 1.23, *p* < 0.001). Despite these within-subgroup differences, the interaction tests for age (*p* for interaction = 0.16) and CKD (*p* for interaction = 0.11) did not reach statistical significance, indicating no formal evidence of effect modification by these two factors.

#### Sensitivity analysis

To assess the robustness of our findings, two sensitivity analyses were conducted.

First, to address potential confounding by urate-lowering therapy, we repeated the main analyses after excluding patients receiving such treatment at admission or discharge. The results remained consistent with the primary findings: compared with the N-N group, the N-H and H-H groups continued to show significantly higher MACE hazards (adjusted HR 1.45, 95% CI 1.06–2.00, *p* = 0.021; and adjusted HR 1.69, 95% CI 1.38–2.07, *p* < 0.001, respectively), whereas the H-N group did not (adjusted HR 0.87, 95% CI 0.69–1.10, *p* = 0.246). The corresponding Kaplan–Meier curves are presented in Supplementary Figure S1, and fully adjusted results in Supplementary Table S1.

Second, multiple imputation for missing baseline covariates (missing proportion 5.1–18.4%) yielded results that were directionally and statistically consistent with the complete case analysis (see Supplementary Tables S2, S3), further supporting the robustness of our conclusions.

## Discussion

This study, using data from the CCA Database-HF, systematically evaluated the associations between static SUA levels and in-hospital dynamic SUA changes with long-term outcomes in 4,000 hospitalized acute HFpEF patients. The key findings are: (1) pre-discharge SUA level, but not admission SUA, was independently associated with the composite endpoint; (2) patients with “N-H” and “H-H” SUA trajectories (indicating in-hospital elevation or persistent elevation) showed higher hazards of MACE; and (3) these associations remained robust after excluding patients receiving urate-lowering therapy. These associational findings extend our understanding of prognostic biomarkers in HFpEF and highlight the potential value of monitoring SUA dynamics during hospitalization, which should be tested in future prospective studies.

### Static SUA and its association with prognosis

SUA has been consistently associated with adverse outcomes in HF (9–12), yet its interpretation depends critically on the timing of measurement. While studies in HF have shown that a single elevated SUA level, often measured at admission or at an unspecified time (14, 15), is associated with worse outcomes, this conventional static approach overlooks the biologically relevant fluctuations that occur during hospitalization. Our study challenges this oversimplification by demonstrating a decisive temporal dichotomy: in the adjusted model, admission SUA was not independently associated with MACE, whereas pre-discharge SUA was strongly and independently associated with MACE. This finding aligns with the growing recognition that longitudinal SUA assessment captures dynamic risk profiles missed by single-point measurements (16, 21), and importantly, it identifies the pre-discharge window as the time point with the strongest prognostic association (11). This distinction underscores a conceptual shift. The admission level likely reflects acute hemodynamic stress and transient volume status—factors that are modifiable with initial therapy. In contrast, the pre-discharge level approximates a patient’s steady-state metabolic and inflammatory burden, which is associated with long-term risk.

### Dynamic SUA trajectories and their association with MACE

The innovative finding of our study lies in defining the association of dynamic SUA trajectories with MACE. Patients in the “N-H” (escalating) and “H-H” (persistently elevated) groups showed higher hazards of MACE compared with the N-N group. This suggests that an in-hospital increase or persistent elevation of SUA is more strongly associated with adverse outcomes than isolated baseline hyperuricemia, corroborating earlier observations in acute HF populations (13, 16, 22).

Traditional single-point measurements cannot capture this dynamic risk. Our study, despite using only two time points (admission and pre-discharge), provides a pragmatic approximation of in-hospital SUA shifts and is the first to demonstrate the prognostic association of such a simplified dynamic stratification in a large Chinese HFpEF cohort, adding a new dimension to risk assessment. On ROC analysis, the optimal SUA cut-off for discriminating MACE risk was 5.6 mg/dL, which aligns with the 5.34 mg/dL threshold from the large URRAH study (23). This provides a potential quantitative reference for hypothesis-generating management strategies.

Consequently, our work reframes the clinical utility of SUA: rather than serving only as a static severity marker, its value lies in its nature as a dynamic biomarker suitable for serial assessment. Even with limited time points, the pre-discharge measurement and the direction of change (escalating vs. De-escalating) offered added prognostic information. Using SUA dynamically, particularly at the transition from inpatient to outpatient care, may enable better risk stratification and suggest a window for closer monitoring (11).

### Potential underlying mechanisms: metabolic and nutritional links

The distinct prognostic associations of admission versus pre-discharge SUA reflect different biological states. Admission SUA primarily captures acute, reversible perturbations during HF decompensation—driven by tissue hypoxia-induced xanthine oxidase activation (24), prerenal azotemia, and diuretic use (25)—limiting its long-term value. In contrast, pre-discharge SUA, measured after clinical stabilization, better reflects the chronic metabolic burden shaped by nutritional and metabolic factors that drive dynamic SUA changes: high purine and fructose intake, insulin resistance, and gut microbiota dysbiosis (26–28).

Recent evidence has expanded our understanding of these pathways. High purine intake increases urate production, while excessive dietary fructose promotes urate generation via ATP depletion (28). Insulin resistance not only reduces renal urate excretion but also actively stimulates urate reabsorption, perpetuating SUA elevation even with moderate purine intake (27). Gut microbiota dysbiosis—characterized by reduced urate-degrading bacteria—impairs extra-renal urate clearance (28), and specific bacterial taxa have been linked to hyperuricemia risk (29). Together, these nutritional and microbial pathways modulate purine metabolism, inflammation, and intestinal barrier integrity (26–28, 30).

Importantly, these mechanisms do not operate in isolation but form an integrated network that underlies the dynamic SUA trajectories observed in HFpEF. High purine/fructose intake and insulin resistance promote hepatic urate production and reduce renal excretion, while gut dysbiosis impairs extra-renal elimination. Collectively, these sustained pathological factors drive chronic inflammation, oxidative stress, and endothelial dysfunction—central to HFpEF progression (12).

Against this background, our findings show that dynamic SUA trajectories—particularly the escalating (N-H) and persistently elevated (H-H) patterns—are more strongly associated with MACE, reflecting underlying adverse biology that single measurements do not capture.

### CKD subgroup: renal function as a potential effect modifier

Critically, renal function may modify the association between SUA and MACE. A 2024 meta-analysis confirmed that hyperuricemia is associated with increased cardiovascular risk, but this relationship is substantially influenced by kidney function (29). A 2025 large-cohort study further reported that HF risk increases linearly with SUA in patients with normal kidney function, whereas this association is attenuated in mild dysfunction and follows a U-shaped pattern in advanced CKD (31).

Consistently, our HFpEF data showed that the significant association between pre-discharge SUA and MACE was restricted to patients without CKD, aligning with prior evidence that hyperuricemia serves as an independent predictor primarily in non-CKD HF populations (32). In advanced CKD, competing risks such as fluid overload and uremic cardiomyopathy may obscure the specific contribution of hyperuricemia (31). Notably, the SUA-to-eGFR ratio has been shown to provide incremental prognostic value in acute HF, suggesting that combining SUA with renal stratification may improve risk discrimination (33).

These hypothesis-generating observations suggest that SUA dynamics may be most useful for risk assessment in non-CKD HFpEF patients, whereas alternative markers may be needed in advanced CKD. Future studies with larger CKD subgroups are warranted to validate these findings.

### Urate-lowering therapy: association and ongoing debate

Sensitivity analysis excluding patients on urate-lowering therapy showed consistent results with the primary findings, further suggesting that SUA is independently associated with outcomes in HFpEF. However, whether SUA lowering is associated with better outcomes remains debated (9, 11, 17). While observational studies in HFpEF have suggested that a ≥ 1 mg/dL SUA reduction is associated with lower mortality (e.g., PURSUIT-HFpEF study (11)), meta-analyses have shown inconsistent associations with cardiovascular death (RR 0.92, *p* = 0.27) (9). SGLT-2i, foundational in HF care (7) and consistently associated with SUA reduction (19, 34), illustrate that multi-mechanistic agents may offer broader benefits. In the EMPEROR-Preserved trial, empagliflozin reduced SUA by 0.99 mg/dL at 4 weeks (*p* < 0.001) (19), and a meta-analysis of 31,535 patients found that every 1 mg/dL reduction in SUA was associated with a 32% lower risk of hospitalization for HF (34).

The metabolic pathways targeted by SGLT-2i—improved insulin sensitivity, reduced oxidative stress, and enhanced uric acid excretion (35)—are also modifiable by diet and nutritional interventions. This overlap shifts our focus from the “whether to treat” debate to identifying high-risk dynamic SUA phenotypes (N-H and H-H groups). We therefore generate the hypothesis that nutritional modulation of SUA dynamics in these phenotypes (e.g., reducing purine/fructose intake, improving insulin sensitivity, modulating gut microbiota) could be tested in future studies to determine whether improving SUA trajectories is associated with better prognosis.

### Nutritional management: evidence and future directions

Building on the hypothesis generated above, this section reviews current evidence on nutritional strategies that may influence SUA dynamics and HFpEF prognosis. To translate our associational findings into a hypothesis-generating framework, we propose a risk-stratified nutritional pathway for HFpEF patients identified with adverse SUA trajectories (N-H and H-H groups). This pathway can be conceptualized as three sequential steps: identification at discharge, targeted medical nutrition therapy (MNT), and longitudinal monitoring.

First, at discharge, SUA trajectories should be calculated from admission and pre-discharge measurements, and patients classified into N-H or H-H groups should be flagged for intensified nutritional follow-up.

Second, for these high-risk patients, individualized MNT should target the three main metabolic drivers of SUA elevation: purine intake, fructose consumption, and insulin resistance (36). Dietary strategies include: (i) a low-purine diet (limiting red/organ meats, certain seafood, and meat broths), supported by a 2025 systematic review of 8 studies (47,879 participants) showing that dietary modification lowers SUA (37); (ii) fructose restriction (avoiding sugar-sweetened beverages and processed foods with high-fructose corn syrup), as specifically recommended by the same review (37); (iii) a plant-based or DASH diet (emphasizing fruits, vegetables, legumes, whole grains, and low-fat dairy), with randomized trial evidence demonstrating a mean SUA reduction of 0.25 mg/dL overall (and 0.73 mg/dL in those with baseline SUA ≥ 8 mg/dL) within 30 days, an effect sustained at 90 days (38, 39); and (iv) adequate hydration (daily water intake >2000 mL, approximately 8–10 cups, unless contraindicated), based on 2025 expert consensus for high-risk hyperuricemia (40).

Third, longitudinal monitoring should include a follow-up visit at 4–6 weeks to reassess SUA, eGFR, BNP, and dietary adherence (18). If SUA reduction from the pre-discharge level is <10%, reinforced nutritional counseling and more frequent monitoring (every 4 weeks) should be considered (41). Thereafter, patients should be followed every 3–6 months alongside routine HF care (42).

When optimal nutritional intervention fails to achieve the guideline-recommended SUA target of <6 mg/dL (18, 40), adjunctive pharmacotherapy may be considered. In HFpEF, SGLT-2i is the preferred option (7, 19, 34). Conventional xanthine oxidase inhibitors (allopurinol or febuxostat) also lower SUA but have shown inconsistent associations with outcomes in HF (9, 11, 17). Therefore, if used, they should be part of a multifactorial management plan and prescribed with caution.

This hypothesis-generating pathway provides a translational basis for future interventional studies testing whether SUA-guided nutritional management improves clinical outcomes in HFpEF.

## Limitations

Several limitations of this study should be acknowledged.

First, this is an observational study; despite rigorous multivariable adjustment and sensitivity analyses, causality cannot be inferred. All findings should be interpreted as associational and hypothesis-generating.

Second, our trajectory classification was based on only two SUA measurements (admission and pre-discharge). This simplification may not fully capture complex in-hospital fluctuations (e.g., multiple peaks or nadirs) and may miss dynamic patterns that require three or more serial measurements. Therefore, our trajectory groups represent a pragmatic approximation rather than a true longitudinal characterization.

Third, the optimal SUA cut-off for MACE prediction (5.6 mg/dL) was derived from this cohort and has not been externally validated. Its generalizability to other populations or settings is uncertain. We therefore suggest that SUA should be used in combination with other clinical markers (e.g., BNP, eGFR, age) for risk stratification, rather than as a standalone test.

Fourth, our analysis did not adjust for several potential confounders due to data unavailability, including: in-hospital loop diuretic dose intensity (cumulative intravenous/oral doses could not be reliably standardized), post-discharge medication adjustments (e.g., initiation or titration of urate-lowering therapy, SGLT-2 inhibitors, or diuretics), lifestyle factors (dietary purine intake, alcohol consumption). These unmeasured factors may bias the observed associations. We have partially addressed exposure by including post-discharge urate-lowering therapy/SGLT-2i/loop diuretic use in Model 5.

Fifth, despite comprehensive covariate adjustment, residual confounding from unmeasured or imperfectly measured variables (e.g., detailed dietary patterns, physical activity, socioeconomic status) cannot be excluded.

Sixth, this is a single-center study conducted in a Chinese cohort of hospitalized acute HFpEF patients. Our findings should be extrapolated with caution to outpatients, other ethnic groups, healthcare systems, or chronic HFpEF populations.

## Conclusion and future directions

Pre-discharge SUA, but not admission SUA, is independently associated with long-term adverse outcomes in acute HFpEF patients. Dynamic in-hospital SUA trajectories (especially H-H and N-H) show stronger associations with MACE than single measurements, suggesting added prognostic value. These hypothesis-generating findings indicate that monitoring SUA dynamics may aid risk stratification. Future prospective studies should validate these associations and test whether nutritional or urate-lowering interventions improve outcomes in high-risk trajectory groups.

## Data Availability

The raw data supporting the conclusions of this article will be made available by the authors, without undue reservation.
