# Supplementary figures and images for: Leveraging dynamic serum uric acid trajectories for risk stratification in hospitalized HFpEF patients
Source: Front Nutr. 2026 Jun 8;13:1802796. doi: 10.3389/fnut.2026.1802796 (PMC13283835; doi:10.3389/fnut.2026.1802796)

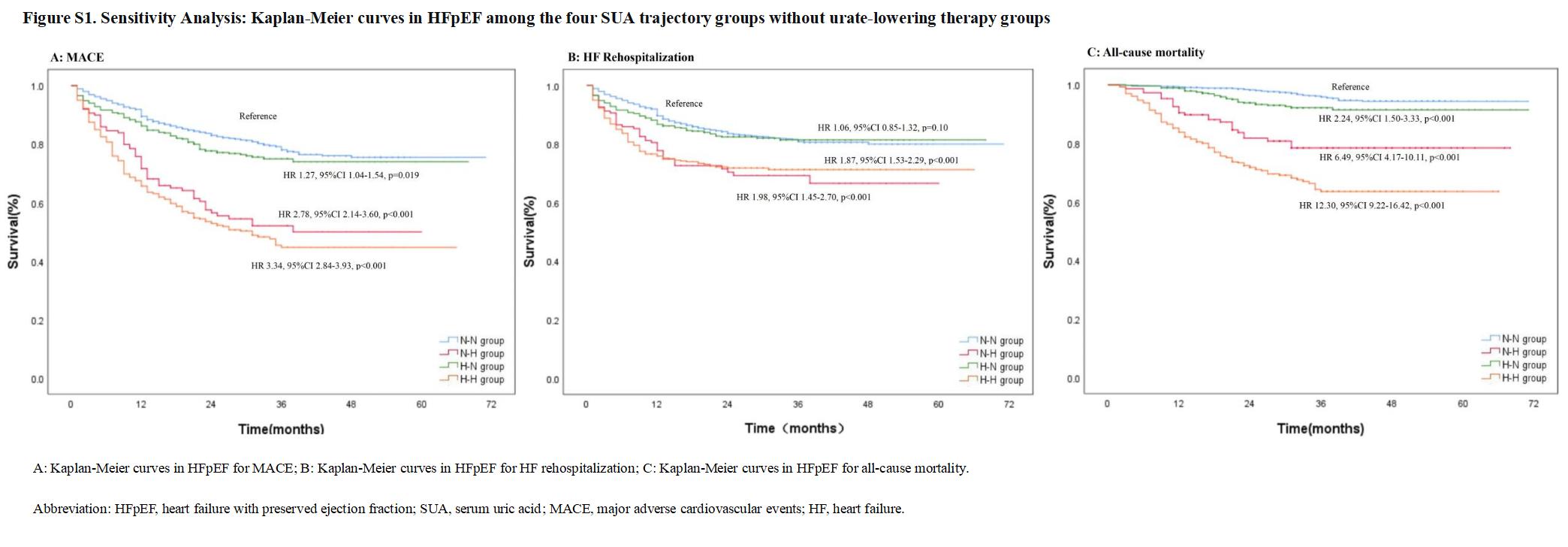

Supplement: Supplementary file 1 [file Image_1.jpg]
