# Supplementary material for: Leveraging dynamic serum uric acid trajectories for risk stratification in hospitalized HFpEF patients
Source: Front Nutr. 2026 Jun 8;13:1802796. doi: 10.3389/fnut.2026.1802796 (PMC13283835; doi:10.3389/fnut.2026.1802796)
Supplement: Supplementary file 2 [file Table_1.docx]

**Supplementary Table S1. Sensitivity analysis: Multivariate Cox Regression Analysis for MACE according to SUA Trajectory Groups without urate-lowering therapy**

|  | N-N group | |  | N-H group | |  | H-N group | |  | H-H group | |
| --- | --- | --- | --- | --- | --- | --- | --- | --- | --- | --- | --- |
|  | HR (95%CI) | p value |  | HR (95%CI) * | p value |  | HR (95%CI) * | p value |  | HR (95%CI) * | p value |
| Model 1 | - | - |  | 2.78 (2.14-3.60) | <0.001 |  | 1.27 (1.04-1.54) | 0.019 |  | 3.34 (2.84-3.93) | <0.001 |
| Model 2 | - | - |  | 2.43 (1.87-3.15) | <0.001 |  | 1.12 (0.92-1.36) | 0.28 |  | 2.95 (2.50-3.48) | <0.001 |
| Model 3 | - | - |  | 1.92 (1.47-2.51) | <0.001 |  | 1.13 (0.93-1.39) | 0.23 |  | 2.29 (1.92-2.72) | <0.001 |
| Model 4 | - | - |  | 1.55 (1.01-2.37) | 0.043 |  | 0.84 (0.56-1.28) | 0.42 |  | 1.45 (1.00-2.11) | 0.051 |
| Model 5 | - | - |  | 1.45 (1.06-2.00) | 0.021 |  | 0.87 (0.69-1.10) | 0.25 |  | 1.69 (1.38-2.07) | <0.001 |

*Compared to N-N group. Model 1: Unadjusted; Model 2: adjusted for demographic factors (age, gender, BMI); Model 3: Model 2+ adjusted for comorbidities (Hypertension, coronary revascularization, diabetes, AF, previous HF hospitalization within 12 months); Model 4: Model 3+ adjusted for laboratory parameters (Hemoglobin, eGFR, BNP before discharge, LVEF, LVEDD); Model 5: Model 4+adjusted for medication history (β-blocker, ACEI/ARB/ARNI, SGLT-2i, MRA, loop diuretic use).

Abbreviations: MACE, major adverse cardiovascular events; SUA, serum uric acid; BMI, body mass index；CAD, coronary artery disease；AF, Atrial Fibrillation; HF, heart failure; eGFR, estimated glomerular filtration rate, BNP, B-type natriuretic peptide; LVEF, left ventricular ejection fraction; LVEDD, left ventricular end-diastolic dimension; ACEI/ARB/ARNI, angiotensin converting enzyme inhibitor/angiotensin receptor blocker/angiotensin receptor neprilysin inhibitor; SGLT-2i, Sodium glucose cotransporter-2 inhibition; MRA, mineralocorticoid recept antagonist
