# Supplementary material for: Leveraging dynamic serum uric acid trajectories for risk stratification in hospitalized HFpEF patients
Source: Front Nutr. 2026 Jun 8;13:1802796. doi: 10.3389/fnut.2026.1802796 (PMC13283835; doi:10.3389/fnut.2026.1802796)
Supplement: Supplementary file 3 [file Table_2.docx]

**Supplementary Table S2. Sensitivity analysis: multivariate Cox regression for MACE according to pre-discharge SUA level using multiple imputation for missing baseline covariates.**

|  | SUA as continuous variable | |  | SUA as binary variable* | |
| --- | --- | --- | --- | --- | --- |
|  | HR (95%CI) | p value |  | HR (95%CI) | p value |
| Model 1 | 1.47 (1.42-1.51) | <0.001 |  | 3.98 (3.32-4.78) | <0.001 |
| Model 2 | 1.42 (1.38-1.47) | <0.001 |  | 3.74 (3.11-4.48) | <0.001 |
| Model 3 | 1.29 (1.25-1.33) | <0.001 |  | 3.05 (2.54-3.67) | <0.001 |
| Model 4 | 1.26 (1.22-1.30) | <0.001 |  | 2.88 (2.39-3.46) | <0.001 |
| Model 5 | 1.25 (1.21-1.30) | <0.001 |  | 2.74 (2.28-3.31) | <0.001 |

*Compared to SUA<5.6mg/dL before discharge. Model 1: Unadjusted; Model 2: adjusted for demographic factors (age, gender, BMI); Model 3: Model 2+ adjusted for comorbidities (Hypertension, coronary revascularization, diabetes, AF, previous HF hospitalization within 12 months); Model 4: Model 3+ adjusted for laboratory parameters (Hemoglobin, eGFR, BNP before discharge, LVEF, LVEDD); Model 5: Model 4+adjusted for medication history (β-blocker, ACEI/ARB/ARNI, SGLT-2i, MRA, loop diuretic use, urate-lowering therapy).

Abbreviations: MACE, major adverse cardiovascular events; SUA, serum uric acid; BMI, body mass index；CAD，coronary artery disease；AF, atrial fibrillation; HF，heart failure; eGFR, estimated glomerular filtration rate, BNP, B-type natriuretic peptide; LVEF, left ventricular ejection fraction; LVEDD, left ventricular end-diastolic dimension; ACEI/ARB/ARNI, angiotensin converting enzyme inhibitor/angiotensin receptor blocker/angiotensin receptor neprilysin inhibitor; SGLT-2i, Sodium glucose cotransporter-2 inhibition; MRA, mineralocorticoid recept antagonist
