# Supplementary material for: Leveraging dynamic serum uric acid trajectories for risk stratification in hospitalized HFpEF patients
Source: Front Nutr. 2026 Jun 8;13:1802796. doi: 10.3389/fnut.2026.1802796 (PMC13283835; doi:10.3389/fnut.2026.1802796)
Supplement: Supplementary file 4 [file Table_3.docx]

**Supplementary Table S3. Sensitivity analysis: Multivariate Cox Regression Analysis for MACE according to SUA Trajectory Groups using multiple imputation for missing baseline covariates.**

|  | N-N group | |  | N-H group | |  | H-N group | |  | H-H group | |
| --- | --- | --- | --- | --- | --- | --- | --- | --- | --- | --- | --- |
|  | HR (95%CI) | p value |  | HR (95% CI) * | p value |  | HR (95% CI) * | p value |  | HR (95% CI) * | p value |
| Model 1 | - | - |  | 2.90 (2.25-3.72) | <0.001 |  | 1.42 (1.17-1.72) | <0.001 |  | 3.12 (2.66-3.65) | <0.001 |
| Model 2 | - | - |  | 2.56 (2.30-2.84) | <0.001 |  | 1.31 (1.21-1.42) | <0.001 |  | 2.69 (2.52-2.88) | <0.001 |
| Model 3 | - | - |  | 2.06 (1.85-2.29) | <0.001 |  | 1.29 (1.19-1.39) | <0.001 |  | 2.13 (1.99-2.28) | <0.001 |
| Model 4 | - | - |  | 1.67 (1.49-1.87) | <0.001 |  | 1.13 (1.04-1.23) | 0.004 |  | 1.83 (1.70-1.97) | <0.001 |
| Model 5 | - | - |  | 1.64 (1.46-1.84) | <0.001 |  | 1.06 (0.98-1.16) | 0.15 |  | 2.01 (1.87-2.16) | <0.001 |

*Compared to N-N group. Model 1: Unadjusted; Model 2: adjusted for demographic factors (age, gender, BMI); Model 3: Model 2+ adjusted for comorbidities (Hypertension, CAD, diabetes, AF, previous HF hospitalization within 12 months); Model 4: Model 3+ adjusted for laboratory parameters (Hemoglobin, eGFR, BNP before discharge, LVEF, LVEDD); Model 5: Model 4+adjusted for medication use (β-blocker, ACEI/ARB/ARNI, SGLT-2i, MRA, loop diuretic use, urate-lowering therapy).

Abbreviations: MACE, major adverse cardiovascular events; SUA, serum uric acid; BMI, body mass index；CAD, coronary artery disease；AF, Atrial Fibrillation; HF，heart failure; eGFR, estimated glomerular filtration rate, BNP, B-type natriuretic peptide; LVEF, left ventricular ejection fraction; LVEDD, left ventricular end-diastolic dimension; ACEI/ARB/ARNI, angiotensin converting enzyme inhibitor/angiotensin receptor blocker/angiotensin receptor neprilysin inhibitor; SGLT-2i, Sodium glucose cotransporter-2 inhibition; MRA, mineralocorticoid recept antagonist
